# Pulmonary Inflammations, Measles, Mumps

**Published:** 1828-06

**Authors:** 


1. Inflammations of the lungs and their appendages, so predominant in the winter, continued to occur, but with mitigated violence, during spring. As the hot weather advanced, we observed the winter coughs of aged persons to subside. On the symptoms and treatment of these maladies, we have spoken so fully, in our last number, that we shall dismiss them with these remarks.

2. Measles. This malady prevailed, generally, throughout the late vernal season; but, although cases of it were met with in every part of the city, it by no means affected all who were liable to experience it. This, doubtless, arose from the qualities of the morbillous matter, whether it might have been diffused through the general atmosphere, or secreted by the bodies of the sick; and, to the same cause we may refer the great mildness of the disease, in almost all who were infected. We heard of no death from this malady throughout the whole season; and, in most cases, it required no professional aid. One of our patients, a woman, nearly 40 years of age, declared that she had suffered the same disease *twice* before. This third attack was of such violence, as to render medical assistance desirable, if not absolutely necessary. That it was measles, appeared, not only from the symptoms, but from the fact, that her three children were infected by her.

3. Mumps. This malady prevailed at the same time with the last; which it resembled, in being extremely mild, and in sparing a great number whom it might have attacked. It generally ran its course, without exciting a call for medical advice. We saw no cases attended with the metastases, which sometimes render it so formidable. In our practice, it offered but a single fact deserving of record. It was this. A little girl of strumous habit, with a well marked scrophulous ulcer beneath the under jaw, laterally, had the disease, in a positive, and rather violent degree, in both parotideæ:—affording an example of the simultaneous presence in the system, and almost in the same parts, of two distinct morbid actions.

[TO BE CONTINUED.]

